# A Case Report of Intrapulmonary Teratoma in the Right Upper Lung Zone in a 35-year-old Female Patient

**DOI:** 10.7759/cureus.3834

**Published:** 2019-01-07

**Authors:** Ahmed A Bawazir, Naif M Alrossais, Yara BinSaleh, Abdulhadi A Alamodi, Abdullah Alshammari

**Affiliations:** 1 Internal Medicine, Alfaisal University, Riyadh, SAU; 2 Surgery, Alfaisal University, Riyadh, SAU

**Keywords:** intrapulmonary, teratoma, mature teratoma

## Abstract

Teratomas are rare germ cell tumors usually found in the gonads. Extra-gonadal teratomas are especially rare, mostly occurring in the thorax. Only a few cases of intrathoracic teratomas have been reported in medical literature and most reported were located in the mediastinum. An additional intrathoracic location for teratoma occurrence is in the pulmonary system, most commonly in the upper lobe of the left lung. In this report, we describe a case of a 35-year-old woman diagnosed with an intrapulmonary mature teratoma found in the right upper lobe. A 35-year-old Saudi female presented with a chief complaint of a three-week history of dry cough. Chest X-ray revealed a right-sided para-pericardial mass in the anterior mediastinum and a significant decrease in the size of the right middle lobe opacity. Subsequently, a chest computed tomography (CT) was performed and revealed a cystic mass in the right lung, which required removal by surgical intervention. The surgery was performed via a thoracoscopic approach and the tumor was excised with no complications. Intrapulmonary teratomas (IPT) usually present with vague and non-specific symptoms such as cough, hemoptysis, and chest pain. It is a rare condition without diagnostic features detected preoperatively, save for trichoptysis which is reported in approximately 13% of the cases. We report a rare appearance of mature IPT in the right upper lung zone which is an unusual location.

## Introduction

Teratomas are rare germ cell tumors composed of one or multiple tissues, such as hair, sebaceous glands, teeth and other tissues derived from germ cells. They are mostly benign and occur most frequently in gonadal organs, namely the ovaries and testicles. However, they can also be found in extra-gonadal areas, especially the thorax [1-2].

Intrathoracic teratomas are usually seen in the mediastinum. However, in some extremely rare cases, intrathoracic teratomas occur within the parenchyma of the lungs [3]. Since its first description by Mohr in 1839 in the literature, only a few cases of intrapulmonary teratomas (IPT) have been reported. It is believed that IPTs are derived from the third endodermal pharyngeal pouch, from which the thymus develops embryonically [3-4]. IPTs commonly involve the upper lobe of the lung, for reasons yet unidentified; patients with IPT usually present with non-specific symptoms such as chest pain, persistent cough, and/or hemoptysis [1-4]. However, in approximately 13% of the cases, patients present with trichoptysis (expectoration of hair) which signifies communication of the teratoma with the bronchus. Surgical resection is currently the sole management method of IPTs and holds a good prognosis in most patients [5].

## Case presentation

A 35-year-old Saudi female presented to her local hospital complaining of a three-week history of dry cough. Her past medical history was unremarkable. Chest imaging revealed a mass in the anterior mediastinum. The patient was referred to King Faisal Specialist Hospital (KFSH) for further workup.

**Figure 1:**
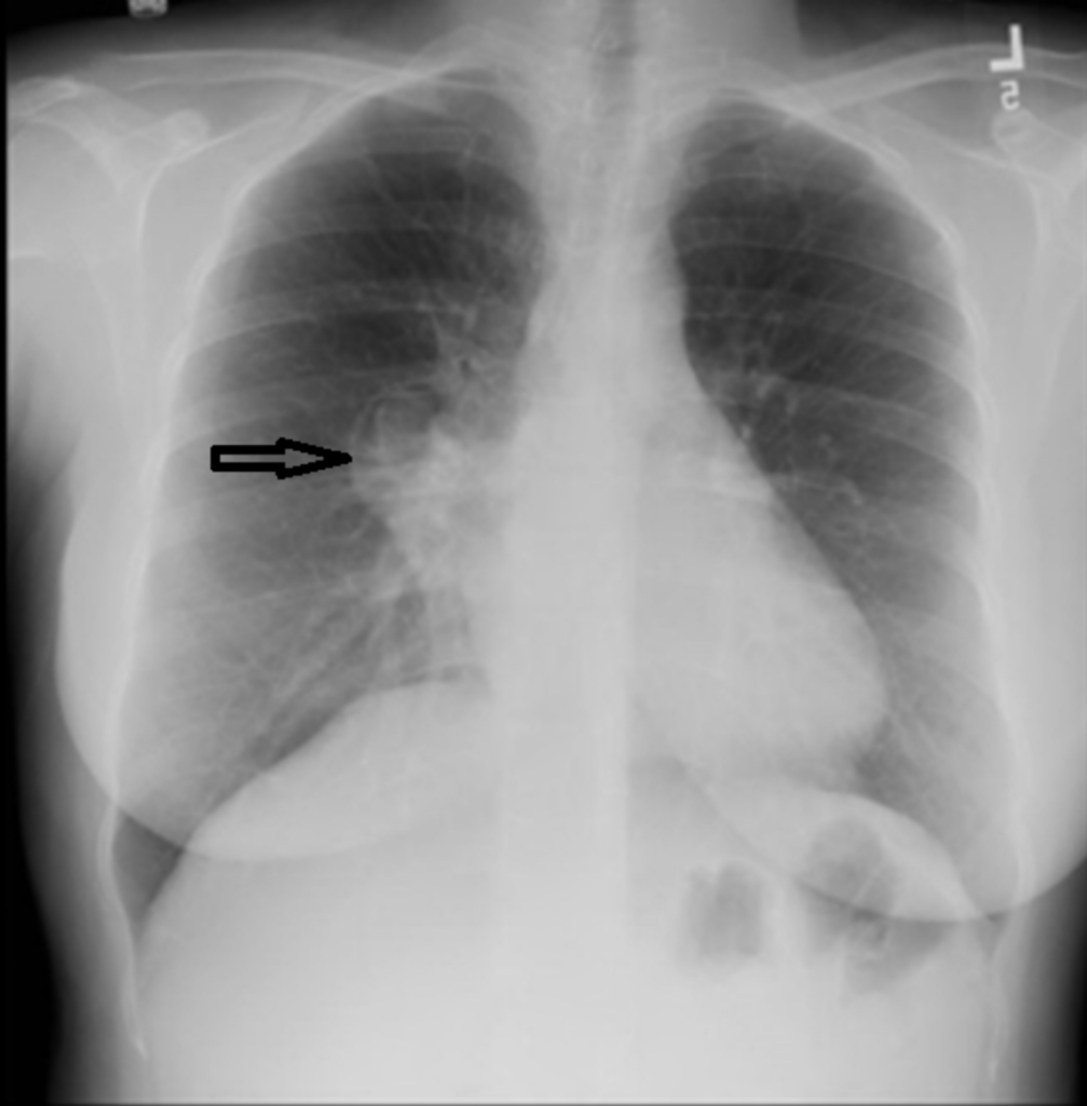
Chest X-ray revealed a right-sided para-pericardial mass in the anterior mediastinum and a significant decrease in the size of the right middle lobe opacity

Repeated chest X-ray revealed a right-sided para-pericardial mass in the anterior mediastinum and a significant decrease in the size of the right middle lobe opacity (Figure 1). No other focal opacities were identified. The cardiac silhouette appeared unremarkable. There was no pleural effusion or pneumothorax.

**Figure 2:**
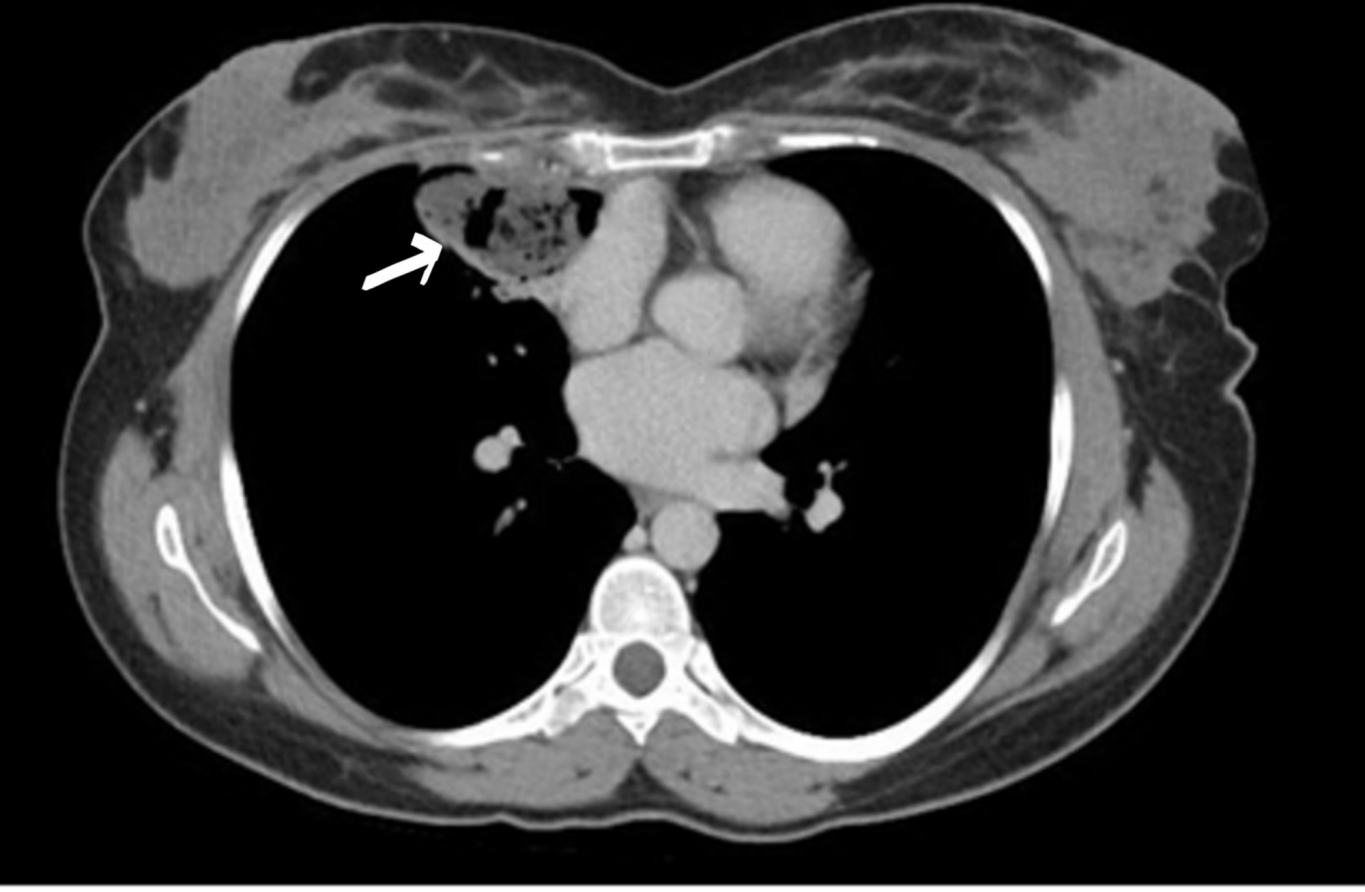
Computed tomography (CT) of the chest revealed a cystic mass in the right upper lung zone

A chest computed tomography (CT) was ordered for the patient, which revealed a cystic mass for which surgical resection was indicated (Figure 2).

The patient was admitted and through a right thoracoscopic approach, the cyst was excised. Intraoperatively, there was significant adhesion of the right lung to the anterior chest wall surrounding the cyst. Dissection was made using diathermy hook and blunt dissection until the lung became almost completely free mainly in the anterior and mediastinal aspect of the pleura. A thick cyst at the anterior segment of the right upper lobe was identified. The cyst was then excised completely by wedge resection and small oozing around the hilum was encountered but controlled by clips. A small incision was made in the anterior chest wall and within was found cheese-like material. A chest tube was inserted in the apex and the anesthetist was asked to inflate the lung, which was done well. The wound was closed in layers, the patient was awoken, and she was sent to the recovery room in good condition. Her postoperative period was uneventful. The retrieved specimen consisted of 64.4 g of lung tissue measuring 14.5 x 6.0 x 3.0 cm. There was a mass opened on the pleural surface measuring 6.0 x 5.0 x 2.5 cm containing soft yellow material. The diagnosis of mature teratoma was then established. The chest tube was removed on the postoperative day two. The patient was discharged on postoperative day three in a good shape. The patient was given a follow-up appointment to the thoracic surgery clinic.

## Discussion

In general, germ cell tumors in the thorax are either secondary tumors, metastasized from the gonads, or developed de novo at the thorax. The most common extra gonadal germ cell tumors in adults are located in the mediastinum [4-6]. IPTs are a very rare germ-cell tumor. By 1996, only 65 cases had been reported in English and Japanese literature, with the first report in 1939. Most IPTs reported are located in the upper left lobe. However, in this case, the IPT was found to exist in the upper right lung lobe [4-6].

Most teratomas are benign and grow slowly. Although teratomas may be present in any age group, IPTs usually affect patients in their first and second decades of life. IPTs can be present in males and females equally, unlike malignant mediastinal germ cell tumors, which affect males more than females [4-8]. Symptoms of IPTs vary according to multiple factors, including their size, location, and tissue type of which they are comprised [7]. The patient, in this case, complained of dry cough, considered the most common symptom of IPTs, alongside hemoptysis and chest pain. Other symptoms of IPTs include trichoptysis, pneumonia, fever, bronchiectasis, abscess, and infection [1-3]. There is no relationship between the size of the teratoma and malignancy [5-6].

Malignant teratomas have a poor prognosis [1-3]. One major complication of IPTs is rupture of the tumor, which some believe to be caused by digestive or proteolytic enzymes secreted by the tumor itself [5-8]. Rupture of the teratoma into the pericardium may cause cardiac tamponade, a medical emergency [4].

The recommended management of benign teratomas is complete surgical excision. Failure to surgically remove the tumor can lead to serious complications including excessive hemoptysis, tumor enlargement, adult respiratory distress syndrome and eventual demise [1-2,5,8].

## Conclusions

IPTs usually present with vague and non-specific symptoms such as cough, hemoptysis, and chest pain. It is a rare condition without diagnostic features detected preoperatively, save for trichoptysis which is reported in approximately 13% of the cases.
